# Doctors’ understanding of their learning and learning needs in Kwazulu-Natal district hospitals

**DOI:** 10.4102/phcfm.v16i1.4375

**Published:** 2024-08-20

**Authors:** Thandaza C. Nkabinde, Andrew J. Ross

**Affiliations:** 1 Department of Family Medicine, College of Health Science, University of KwaZulu-Natal, Durban, South Africa

**Keywords:** medical doctors, learning, learning needs, rural, district hospitals

## Abstract

**Background:**

Medicine is a self-regulating profession. Doctors must learn how to self-regulate to keep up-to-date with evolving health care needs. This is challenging for those working at District Hospitals (DHs) in rural settings, where limited resources and understaffing may compound a poor approach and understanding of how to become a self-directed learner.

**Aim:**

To explore perspectives of doctors working in rural DHs, regarding their understanding of learning and learning needs.

**Setting:**

This study was conducted in Bethesda and Mseleni DHs, in rural KwaZulu-Natal.

**Methods:**

This was a qualitative study. Data was collected through 16 semi-structured interviews and non-participatory observations.

**Results:**

Four major themes emerged: “Why I learn,” “What I need to learn,” “How I learn,” and our learning environment.” This paper focussed on the first three themes. Doctors’ learning is influenced by various factors, including their engagement with clinical practice, personal motivation, and their learning process. Deliberate practice and engagement in reflective practice as key principles for workplace learning became evident.

**Conclusion:**

In rural DHs, doctors need to take a proactive self-regulated approach to their learning due to difficulties they encounter. They must build competence, autonomy, a sense of connection in their learning process, thus recognizing the need for continuous learning, motivating themselves, and understanding where they lack knowledge, all essential for achieving success.

**Contribution:**

This article contributes towards strengthening medical education in African rural context, by empowering medical educators and facility managers to meet the learning needs of doctors, thus contributing to the provision of quality health care.

## Introduction

Medicine is considered a self-regulating profession, making it subject to social contracts that require certain standards are met in practice by health care professionals.^1^ No matter where medical doctors work, they all are expected to possess professional skills that are up to date and tailored towards the evolving health care needs of the people they serve. Upon graduating, medical doctors must learn how to become self-directed learners or else run the risk of becoming obsolete because of the accelerated growth of knowledge and disease profiles.^2^ This means in the working environment, doctors are expected to willingly engage in ongoing professional development activities to maintain learning and professional competence^3,4^ to provide context-relevant, responsive, quality health care. Doctors who are skilled and motivated and who understand the need for ongoing learning are critical for achieving universal health coverage, which is an aspiration of the South African Government.^5^

Learning to become a self-directed learner is a challenge for many doctors, particularly for those who find themselves working at district hospitals (DHs) in rural settings, where limited resources and understaffing may compound a poor approach/understanding of how to become a self-directed learner. Many rural DHs have a flat authority structure, in that everyone works as medical officers or clinical managers, with insufficient specialist consultants to provide supervision, teaching and oversight of junior staff. This places the responsibility for self-directed learning on the rural hospital doctor.

District public sector hospitals in South Africa, unlike regional hospitals, are generalist facilities, with approximately 200 beds being staffed by 10–15 medical officers. These hospitals offer a referral service to the primary health care clinics in their catchment area and provide out and inpatient services. Doctors working at these hospitals are expected to provide generalist services and, when on call, would be expected to see and manage patients presenting with various conditions. Because of the wide range of clinical conditions, it is essential that doctors learn to continually engage with ways of learning and professional development to enable them to provide quality services. Although the need for continuous professional development is widely acknowledged, identifying optimal implementation strategies for doctors working in rural environments, many of whom feel academically isolated, remain a challenge.^5^

There is limited literature, especially in a South African context, on how doctors working in a rural DH environment understand their learning and learning needs. The aim of this study was to explore the views of medical doctors working at rural DHs in the province of KwaZulu-Natal (KZN) in South Africa (SA) on how they understand learning and their learning needs. These insights might help medical educators and facility managers better meet these learning needs, which might contribute to the provision of quality health care.

## Research methods and design

This was a qualitative study conducted at two DHs in northern KwaZulu-Natal, these being Bethesda and Mseleni hospitals (MH). Bethesda Hospital (BH), situated in the small village of Ubombo on the Lebombo mountains, has 230 beds and serves a population of 100 000 people who live in an area of approximately 1500 km^2^. On average, BH has 10–15 doctors, who spend approximately 2 years employed at the hospital, with a small cohort having been there for 5 years or more. Mseleni hospital, situated in Mseleni village, has 219 beds and serves a population of 90 000 people with 10–15 doctors at any time, most only staying for up to 2 years at the hospital. Purposive sampling was used to select eight participants from each hospital, the focus specifically being on information-rich individuals who would help explore the topic.

Data generation involved interviews and non-participatory observations. Firstly, 16 semi-structured interviews were conducted with all participants (eight from each of the two hospitals). An interview guide was developed from social cognitive theories (or social learning theories) by Albert Bandura,^6^ which was influenced by a situated model of self-regulated learning.^7^ Secondly, to support the semi-structured interview findings, participants consented to non-participatory observations at morning hand-over meetings, continued medical education sessions, grand ward round teaching sessions and paediatric morbidity and mortality meetings at each hospital to observe how the doctors engaged with learning. Field notes were taken during the observations to record how they engaged with different learning scenarios, their colleagues and the learning environment. These assisted in understanding and interpreting interview findings and questions formulated for the focus group discussions.

All interviews were audiotaped, downloaded onto a password-protected laptop and transcribed verbatim. Codes, categories and themes emanating from the data were identified. Throughout this process, there was continuous recording of memos, thus ensuring transparency and trustworthiness of the analysis process. A coding system (BH1-8 and MH1-8) for quotations was used to identify the participants to the researcher but to protect their identity on reporting the findings.

### Ethical considerations

The research proposal was approved by the University of KwaZulu-Natal Biomedical research Ethics Committee (Ethics number: BE 107/19). Permission was also obtained from the KZN Department of Health research committee, and the two individual hospital research committees. All participants signed informed consent prior to participating.

## Results

Sixteen doctors (8 from BH and 8 from MH) participated in the study. At BH, the male to female ratio was 6:2, with five being black African and three being white (1 African white, 2 foreign) and three being from the local Jozini municipality. Six held a South African MBChB qualification, of whom one was doing their community service year, and two had a foreign qualification. At MH, the male to female ratio was 5:3, with four being black African and four being white (2 African white, 2 foreign white) and three being from the local Umhlabuyalingana municipality. Five had a South African MBChB qualification (three were doing their community service year), and three were foreign qualified.

Four major themes emerged from the data analysis: Why I learn, what I need to learn, how I learn and our learning environment. For this paper, only the first three themes will be presented, as the learning environment and theoretical frameworks will be described in subsequent articles. For each of the themes, subthemes were identified, with direct quotes being provided to support their understanding.

### Theme 1: Why I learn

The findings of this category are divided into six subthemes, as indicated in Table 1, followed by a discussion.

**TABLE 1:** Theme and subthemes for ‘Why I learn.’

Theme	Subthemes
1. Why I learn	1.1. Identification of gaps – I do not know everything 1.2. To provide quality patient care – beneficial to the patient and provider 1.3. To improve practice 1.4. For continuous self-development, independence and sense of competence 1.5. A sense of responsibility – patients are depending on me 1.6. I have fear! – a driver or hindrance to learning

#### Theme 1 findings

The participants were asked to engage with the topic of learning and learning needs, define what this means to them and elaborate on why they feel the need to learn. From the participants’ responses, six subthemes were identified (see Table 1) and are discussed further.

**Subtheme 1: Identification of gaps – I do not know everything:** Overwhelmingly, participants learnt because they do not know everything and identified gaps in their knowledge and skills brought on by not feeling comfortable in their clinical practice:

‘When I got to this hospital, I was expected to do calls. And then I realised all my shortcomings, and my needs then were highlighted, … I felt under pressure (so) I needed to upscale my knowledge and skills.’ (BT2, MR, F2)

The lack of experience of working in a rural hospital highlighted their need to learn more, given that they were expected to function as a generalist. One participant noted that he needed:

‘[*T*]o identify gaps in (your) learning and then (you) need to fill those regardless of what stage of his career he found himself in.’ (MT4, LH, F5)

Participants understood that their learning and learning needs were dynamic and would change over the years as:

‘[*M*]edicine is always developing, so even if you knew everything 10 years ago, you still would not know everything now.’ (MT6, VF, M9)

This meant that as gaps in their knowledge were being continuously identified, they need to constantly engage in the process of learning. Junior clinicians had learning gaps, which were different to their senior colleagues, with many of these learning needs having emerged from their internship years, while their seniors felt their gaps were linked to the responsibilities they had taken on:

‘As a junior, my learning needs are varied and many have come out of internship, where we are sort of equipped with the bare minimum.’ (MT3, BA, F4)

‘As I am growing for a number of years in this environment, I’m also now regarded as one of the seniors, so I feel that I need to take that responsibility on.’ (BT8, NT, M3)

**Subtheme 2: To provide quality patient care (Beneficial to the patient and the provider):** Participants were clear that they needed to engage in effective ongoing learning, to provide quality healthcare, as:

‘… (learning) … is a continuous way of developing your skills, to actually take good care of your patients over time.’ (BT4, TN, M4)

Developing their clinical skills and filling their knowledge gaps helped to meet the needs of the patient, and ensured that they could perform their job safely and efficiently, which also contributed to their work enjoyment as:

‘(Continuous professional development) … is more than just doing your job better. It’s also about job satisfaction and the enjoyment of work itself.’ (BT1, KG, M1)

There was an understanding that the learning and care they provided, needed to be patient-centred and relevant to the patient’s needs. This meant they needed to engage in active introspection and reflection during the learning process and be willing to change themselves should there be a need, which would help foster service delivery to the patients:

‘We need to learn how to use ourselves, and our personality to serve the patient and how to change our personality where it’s a hindrance.’ (MT6, VF, M9)

**Subtheme 3: To improve practice:** In order to improve their clinical practice, participants were mindful that they needed to continuously question their current clinical practice, thus ensuring they were not stagnant in the way they treated patients. In this way, they actively engaged in a self-regulatory process, which is important to think about, as learning and learning needs change with time:

‘I suppose … you have to keep up to date with why you are doing something, is this the only guideline that’s available? You mustn’t become reliant on just this single thing and becoming like a puppet just doing whatever, because then anyone can be a doctor, but you have to understand the reasons behind things and why you are doing things.’ (MT1, VV, F3)

Participants recognised that professionals needed to be willing to learn and relearn certain skills and fill knowledge gaps as they are identified and must understand the importance of growth from one phase to the next as a clinician. Professionals should be constantly asking themselves:

‘What do I need to (learn) to advance myself on a professional or a personal basis?’ (BT1, KG, M1)

They also recognised the need to get into the habit of:

‘[*A*]rming yourself with as much information as you can (so) that you don’t think you’re going to make a mistake some where.’ (MT5, GU, M8)

**Subtheme 4: For continuous self-development/independence/a sense of competence:** Participants recognised that by improving their clinical practice, they were engaging in a process of self-development, which led to greater independence and competency. It also meant being willing to identify blind spots and to take action to fill them:

‘Stepping back a bit, there are the day-to-day needs …, you want to be able to work independently and do your job as best as you can.’ (BT5, CK, M5)

‘We learn every day, … every day when you learn something you get to understand, oh, so I didn’t know that, then you see a gap and say, oh, there was a gap, let me go and fill it.’ (MT5, GU, M8)

The participants were motivated to learn to meet the changing needs of their patients and that:

‘[*L*]earning has to do with what you want to attain, how far you want to go and where you see yourself (in the future).’ (MT5, GU, M8)

Others aspired to work in rural hospitals as they were looking for ways to:

‘… be a better generalist’ and recognised that a generalist ‘functions better in district level rural health settings.’ (BT2, MR, F2)

**Subtheme 5: A sense of responsibility – Patients are depending on me:** The sense of responsibility for their patients was a stimulus for learning for many of the participants. Working in rural hospitals, doctors are often required to take on responsibilities over and above the clinical management of their patients and to cope in less-than-ideal circumstances. This environment, although stressful and challenging, presented opportunities for learning that could only be acquired through experience and which may serve their careers in the future. However, one doctor spoke of the stress of working in isolation, having to cope without support, and the need to learn to enable him to rise to the challenges he faced. These experiences stimulated his learning because of the sense of responsibility that he felt for the patients under his care. When reflecting on those experiences, he was able to acknowledge the challenges of working under less-than-ideal circumstances, and having to take responsibility was an important stimulus for his learning:

‘When I came back here patients were dying, it was chaos, there was no one else around. Often I would be the only doctor left in the hospital.’ (BT1, KG, M1)

‘Although I knew I wasn’t doing the best job that could be done, I knew that I was doing the best job that I could do, and that situation created a learning environment, coz then I want to advance myself, I want to help patients – that’s what I’m here to do.’ (BT1, KG, M1)

**Subtheme 6: I have fear! (A driver or hinderance to learning):** Rural environments can be challenging to work in, with some health care providers having to operate outside their comfort zone. Some thrive in this environment and recognised the potential that this has to influence professional growth:

‘One of the reasons for applying to come here was because you are in a rural hospital, and would be forced to learn and do procedures that I am not necessarily comfortable with and just kind of stretch my skills.’ (MT1, VV, F3)

This approach may not be true for everyone, with some noting that their sense of being ill-prepared had the potential to induce fear, which can affect their ability to learn. Fear of failure motivated some to acquire more knowledge and skills, while for others, it was so overwhelming that it resulted in inactivity and not facing the challenge by better equipping themselves to meet these challenges:

‘I think not just fear of failure, but emotions in general impacts our learning. I think fear does drive learning to some degree. I don’t know if it’s always as productive as other incentives or other drivers, but yes, we are all driven by primitive drives. Even in this environment, there’s different reasons why people want to learn, and fear of failure or looking stupid and being a disappointment is one of them. But fear can also make you shy away from learning as well. You will often see that in this setting that people who shy away from areas they are uncomfortable with and avoid certain situations or a learning need because of the fear.’ (BT1, KG, M1)

#### Discussion

The respondents highlighted various reasons why they learn and that improving their clinical practice by engaging in self-development leads to greater independence and competence, enabling them to provide better quality health care. There is a need for doctors to learn through self-assessment and engaging in practices that lead to improvement in knowledge and clinical care.

There is evidence in the literature that medical doctors are motivated to learn either by specific problems posed as questions about particular patients or by general problems that highlight gaps in their skills or knowledge.^8^ These specific or general problems need either semi-structured or formal approaches to address them and ensure that learning takes place.^8^

Providing high quality care for patients is an important professional goal for many, and to achieve this, doctors need to keep learning, as a deliberate and focused practice, which then leads to improved performance.^9^ By aiming to deliver high quality health care, the medical doctors’ everyday experiences contributed to their professional development and learning.

Anxiety or fear can often be associated with negative emotions, but research done among doctors working in a rural environment has shown that it can also be stimulating for the development of clinical acumen for individuals engaging in a particularly challenging environment and can be viewed as positive emotion for learning.^10^

### Theme 2: Theme and subthemes for – ‘What I need to learn’

The responses for this theme were divided into five subthemes, as indicated in Table 2, with the results being followed by a discussion.

**TABLE 2:** Themes for ‘What I need to learn.’

Theme	Subthemes
2. What I need to learn	2.1. About common general and emergency medical conditions 2.2. Skills needed to function as a generalist in a rural environment 2.3. Becoming and being a senior to others 2.4. Being a mentor to others 2.5. Understanding community and cultural norms

#### Theme 2

As mentioned previously, it is important that medical doctors continuously update their knowledge and skills to be able to provide appropriate, context-specific care to the patients that they see. Adult learners are often driven to learn by real-life experiences that expose their knowledge gaps. For these experiences to lead to learning, they need to be able to construct knowledge and meaning from these encounters, a subject known as experiential learning.^11^ To better understand their learning needs, participants were asked to comment on what they perceived to be their learning needs, to highlight how they centralised learning around them.

**Subtheme 1: (learning) About common general and emergency medicine conditions:** Half (8 of 16) of the participants were in their community service year and therefore regarded as junior clinicians or were medical officers who had spent a year or less in that hospital. Regarding what they felt they need to learn in this environment, most junior clinicians focused on gaining clinical knowledge and skills in the various disciplines they were exposed to in a DH setting. A number of them felt overwhelmed by the amount of learning they needed to do:

‘As a junior doctor there is a lot of learning to be done.’ (MT8, GA, M11)

‘To me, learning needs, especially coming here, I was like a fish out of water to start with, in terms of the environment, what clinical and practical knowledge I need on the job.’ (BT3, CB, M2)

Although the areas identified as learning needs were broad, there was an emphasis on emergency care, as that could lead to death if not managed correctly. Participants also felt the responsibility that comes with being a doctor and the need to act even while waiting for help:

‘[O]h, let’s say you’re first on call and there’s a Basic Life Support or an Advance Life Support emergency that needs to be done, obviously you will call the second on call to help you but meanwhile you are a doctor who’s there. You need to be team leader at that time. You can’t just wait for someone to come help.’ (BT6, NM, F1)

‘So, it’s basically emergency medicine … because you are too far away from referral institutions.’ (MT7, ML, M10)

Some participants indicated that multiple factors influenced what they learnt, such as where they were in their careers, or having moved to a new work environment. Becoming or being considered a senior because of their clinical knowledge or years spent in this rural environment meant that they had to take on more non-clinical responsibilities, which required them to learn about managerial and clinical governance:

‘I suppose my learning needs initially were just to try and refresh cases that I haven’t dealt with in quiet some time … and obviously then just building on that and re-learning procedures that I hadn’t done in a long time.’ (MT1, VV, F3)

‘An opportunity arose to take on a management position at the hospital and that in itself was a steep learning experience. Because now not only do I have that clinical knowledge and the procedural knowledge that I felt more comfortable with, and felt I could make a difference with, but now I suddenly realized there was a lot more managerial stuff that I needed to learn.’ (BT1, KG, M1)

**Subtheme 2: Skills needed to function as a generalist in a rural environment:** The participants indicated that practising as a generalist in a district level hospital meant they must have the skills to attend to any and every patient who presented for healthcare. This meant that they needed to broaden their knowledge and skills base and be competent as a generalist. For some, this proved to be challenging, as it required them to think beyond just working in a single discipline or department, given that most undergraduate training in South Africa is discipline-based:

‘Well, I think for me to be a doctor, able to manage the scope of patients you see in a general environment … I need to broaden my knowledge and my skills to a point where I can manage a broader scope of patients.’ (MT3, BA, F4)

Working as a generalist in rural areas was not limited to hospital care but included knowing and engaging in primary health care services and understanding the community they serve. This meant that they need to engage with the broadness of medicine practised in such an environment, without having specialists on site to advise them. While some participants may have preferred certain aspects or specialities of medicine when they were not working in a rural environment, in a district hospital they had to learn to engage with different clinical scenarios, regardless of their preferences:

‘The scope of rural medicine is very broad, we don’t have specialised departments like in other hospitals.’ (MT7, ML, M10)

‘Although some people prefer surgery or paediatrics, here you have to know everything, to attend to every case, even when you personally do not like those cases, you’re still expected to see and manage them properly.’ (MT7, ML, M10)

**Subtheme 3: Becoming and being a senior to others:** Being considered a senior in a clinical environment comes with many expectations and responsibilities. These forced individuals to learn more quickly and contributed to building confidence for future independent practice:

‘So, you know when you mature into certain positions at work, then certain people expect you have some background knowledge about certain things.’ (MT4, LH, F5)

‘[*Y*]ou start to think, OK, well I am going to be a senior, alone one day, and there is going to be a lady needing, let’s say a hysterectomy, and now that is a skill that I haven’t been able to learn but I have an opportunity to learn because there is someone that can perform such a skill, and I just feel like maybe I am running out of time to learn the skill from this particular individual while they are still around.’ (MT4, LH, F5)

Learning how to become a senior was identified as a critical learning need by some participants, as it forced them to learn how to teach and supervise others on the clinical platform and to think beyond clinical practice into health systems matters at district, provincial and national levels. It also meant becoming sensitised to colleagues’ learning needs, being aware of and giving them opportunities to learn, thereby allowing them to progress professionally. However, this is made difficult as many rural environments have no formal programmes that would help equip them to become a senior:

‘Having to be sensitive and realise what are other people’s learning needs are, and taking into account the bigger picture of the objective and what district healthcare should be all about, and what they wanting to achieve as a hospital, district or at national level.’ (BT2, MR, F2)

‘I think professional progress requires somebody who is prepared to recognise that you have the skills. And that’s becoming more and more necessary in order to be able to practice Medicine … But I’m also aware that a lot of doctors coming to us needing that, and I’m aware that they need to have the opportunity to do that. I’ve been quite keen that there would be opportunities to do diplomas and such like for doctors in these settings, for their career progression.’ (MT6, VF, M9)

**Subtheme 4: Being a mentor to others:** Mentorship is closely associated with seniority, and for individuals to grow professionally, they often need to be mentored to experience learning in action from their colleagues. However, this process needs intentionality, initiative and structure in order for it to be effective and for all parties to be involved in the process. Participants identified mentorship as an area that needed to be improved, as many had experience of having good mentors around them in their junior years:

‘For one to be sure that they learning, whoever is supposed to learn must see the individual who is teaching them, what he’s doing, so they can identify how things are done.’ (BT7, MM, M6)

‘I’ve actually spoken to some people in management about it and said we can start a mentorship program within the hospital, where a manager … chooses two, three people to say I am grooming you to be a manager one day …’ (MT4, LH, F5)

A senior participant indicated their availability for junior colleagues to learn from them as a mentor and role model and constantly asked themselves:

‘Am I accessible enough for people who want to learn from me, and who want to draw knowledge from me … Can they learn from the way that I do things, how I practice Medicine?’ (MT4, LH, F5)

Another participant felt it was important to assess the current context and evaluate how it will change in future, especially when in a year’s time, junior clinicians would be expected to be senior and mentor others, which makes this a potential area of learning need:

‘[*B*]ecause maybe early next year you’ll be doing a second on call (as a senior), you’ll be the one who’s actually mentoring someone or teaching someone to actually do that certain skill. So those are the things that you sort of see yourself learning and being pushed, or push yourself to do because you have to.’ (BT6, NM, F1)

**Subtheme 5: Understanding the community and cultural norms:** Many participants identified the need for community engagement, as it would help them to better understand the clinical needs as well as the cultural context and practices, which could affect the way they practise medicine. The differing cultural approaches to health and healing in rural areas highlighted the need to learn how to engage with the community with sensitivity, so as not to undermine their beliefs and practices:

‘I think one thing that I would have wanted to learn, perhaps much earlier on, is to be able to deal with and learn how to interact with the community and understand them, where they’re coming from. To learn how to interact with them in a way that is sensitive, that doesn’t undermine their beliefs, their culture. I don’t think we’re trained in those sorts of things but we come across them every day, and how we handle them can actually impact a lot on the outcome and relationship with that community, or with that particular patient. It can determine whether the patient will come in or not. It can determine whether the patient trusts the system.’ (BT2, MR, F2)

A number of those who had worked at the hospital for a few years had become involved in community outreach initiatives and noticed the potential for impacting positively on various health issues. They highlighted the importance of engaging with how social determinants affect the provision of health care and encouraged clinicians to not be hospital-centric in their approach, but to consider ways of engaging with communities:

‘[*I*]f you’re here a few years and you really get involved with the community – you start to think about how to engage in community activities more and more … there seems to be more opportunities out there, to be more effective in the community.’ (BT5, CK, M5)

The responses indicated that being from the community and now practising medicine in the same community was beneficial for the individual, the hospital and the community, as it enhanced the provision of culturally sensitive good quality care by people who were known and trusted. They understand community practices, giving further depth to the consultations and engagements between the provider of care and the patient, this being important when having to negotiate care plans that cut across cultural differences:

‘It really helps being from this community, because I understand where people are coming from when they talk about cultural aspects.’ (BT7, MM, M6)

‘Coming from the same environment, speaking exactly the same language as the people of the community and understanding the challenges of the community is a benefit, because in my conversations there are things I can negotiate with the patient. It helps me understand the community dynamics, which is a benefit to the hospital as well.’ (BT4, TN, M4)

This was not the case for the junior doctors, who felt they needed to focus on acquiring clinical knowledge and skills before considering community involvements. Foreign qualified doctors, and South Africans who did not speak the local language faced an additional challenge of language barriers, impacting their consultations and interactions with patients:

‘Particular in this environment, is the language barrier, it definitely would be a very big help if I could be fluent in the local language … so that I can communicate with the patient, or even just connect with the patient, so they can feel I am making an effort to meet them in their own language, or greet them at least.’ (MT1, VV, F3)

#### Discussion

From the subthemes described, learning needs ranged from emergency clinical care and working as a generalist, to clinical governance, teaching and mentorship to community engagement, all depending on the level of career progression of the participants. The more junior the participants, the more their learning needs aligned to clinical aspects, and, as they progressed in this environment, so the non-clinical aspects become more important. Clinical knowledge and procedural skills gaps were often identified in daily clinical practice and were common, consistent with other studies found in literature.^12,13,14^

These findings support a concept documented in literature as ‘transitioning’, which is when individuals move from a feeling of dependency to independency as they actively develop their professional knowledge, skills and identity. Some authors warn that transitioning can negatively impact professional learning if there is no adequate support, thus hindering the progression to ‘expert.’^14^

Rural generalist medicine has been defined by the 2014 Cairns consensus statement on rural generalist medicine as a broad scope of rural medical care that covers comprehensive primary care, hospital emergency care and advanced skill sets.^15^ However, there are many definitions of generalist medicine, and these depend on the context, nationality, background and discipline of the authors.^16^ There is no better place for doctors to learn to practise as a generalist than in rural hospitals. These environments offer good generalist experiences,^17^ as one is required to treat everyone that comes through the door regardless of the condition. The rural medical doctor must possess clinical courage, an inner debate where the needs of the patient and the extent of prior training and experience intersect.^18^ This is particularly true for junior doctors, as they have to learn to deal with greater responsibility, as they often need to operate outside their comfort zones.^19^

The development of teaching skills is a core competency in many postgraduate medical training programmes.^20^ Although not a formalised programme, many doctors working in rural environments engage in ‘peer assisted learning’, a concept well established in medical education,^21^ where individuals help others to learn, and learn themselves in the process.^22^ This type of learning has been happening for many years in medicine, including in rural environments. It is important that greater emphasis is made to formalise this process for those who are not involved in postgraduate programmes but who find themselves practising in rural district hospitals to ensure they are confident in their ability to assist others to learn.

Being a mentor is described as ‘an experienced person who goes out of their way to help a mentee set important life goals and develop the skills to reach them.’^23^ According to the social learning theory,^24^ individuals tend to imitate the behaviours of their seniors (mentors), as they often respect and admire them.^25^ In order for an individual to transition from being a mentee to an expert, they often need to be a part of a good mentoring supervision process,^26^ as this process ensures a space that is protected, but at the same time allows the learner to make mistakes, face difficulties and celebrate their accomplishments as they learn. Formalisation of this process in a rural context appears to vary, depending on the attitude of the senior staff, and needs intentionality and effort, according to the participants in this study, as it is critical for ensuring ongoing learning for all.

### Theme 3: Theme and subthemes for – ‘How I learn’

For individuals to be successful in their learning, they need to understand how they learn, which means they need to learn how to critically reflect on their experiences and develop a process of self-criticism and introspection. The four subthemes related to ‘How I learn’ are listed in Table 3.

**TABLE 3:** Theme 3 findings – How I learn.

Theme	Subthemes
3. How I learn	3.1. It all starts with me (Personal motivation) 3.2. Introspection and reflective practice 3.3. Resources to learn 3.4. Formal and informal learning

#### Subtheme 1: It all starts with me (Personal motivation)

Participants in this study identified that learning in a rural environment away from traditional learning tools, is difficult and often requires them to have a persistent attitude of seeking out knowledge by trying different approaches, which one participant described as follows:

‘[*I*]ts more like trying out different doors, so I will try a door until I find one that I feel more comfortable walking through.’ (MT1, VV, F3)

As there are many barriers that need to be overcome, it is important that individuals have self-motivation and self-drive to persevere in such a setting. Having a conscious sense of curiosity makes the process of engaging with learning easier, as it has the potential to broaden their thinking and approach to self-regulation:

‘One must have an insatiable curiosity for learning, and often have periods of time when one deliberately reads around subjects that one is not necessarily dealing with at the time … This way you learning about things you don’t know so you can recognize them when they turn up, because a lot of Medicine is recognition, and you only recognise things when you know they exist.’ (MT6, VF, M9)

Self-drive and self-motivation are important, given that the doctors come from different backgrounds and have had varying learning experiences. Some participants compared themselves with their peers in terms of knowledge and skills, while others would approach other clinicians, and make it known that they needed help and were intentional about their learning:

‘As a person who trained in Cuba, when I came back I tried to figure out where do I lack (knowledge and skills) and how can I make it to the standard of my fellow South Africans who studied here … that background gave me more power, more energy to say you know what, push to learn, don’t be at the back.’ (MT5, GU, M8)

‘(You need to) make it known to people you are working with so they can help you, so I made it known to the manager by saying I would love to be exposed in certain area.’ (MT2, GW, M7)

#### Subtheme 2: Introspection and reflective practice

Introspection and engaging in reflective practice were important to participants, particularly in how they approached their learning. As doctors, some project into the future by setting goals as to what they would like to achieve or where they would like to be in their careers:

‘Everything that I do I like to associate it with what I would like to do in future.’ (MT5, GU, M8)

Introspection and being a reflective practitioner are closely linked to self-drive and motivation, and for some participants, this went beyond learning in the clinical field to include other areas of their life. This finding highlighted that individuals were not only focused on their clinical learning, but rather recognising that how they learnt had multiple influences that they needed to consider:

‘I started reflecting, and it wasn’t just reflecting on work related things, it was about reflecting on who I was as a person, who I wanted to be. Reflecting on what kind of a mother I wanted to be for instance. What kind of a wife I wanted to be for my husband? And then you go into personality assessments – who am I? How do I relate to the next person? what makes me tick? what makes me happy? what makes me angry? what’s going to propel me into the future.’ (MT4, LH, F5)

#### Subtheme 3: Resources to learn

Rural environments are known for having limited resources to facilitate professional development, specifically those things that could aid in continuous learning. The availability of hard copy clinical guidelines appeared not to be a problem, as did access to online journals in a range of fields, with doctors continuously engaging with them as part of their learning process. Participants who had access to internet services recognised that ‘the internet is a big part of education when you’re stuck in the middle of nowhere, having access to internet makes a huge difference’ (BT5). Access to these services enabled them to use online and printed resources to aid their learning, with some participating in online learning courses:

‘I find myself often looking at guidelines, trying to look at what’s the best research … especially those that I haven’t been exposed to very much.’ (MT3, BA, F4)

Although information was noted as being more accessible because of the internet, some participants raised concern over the associated data costs, which are usually the responsibility of the individual engaging with learning. While written text provided important information, the growing use of videos as learning instruments was a considerable help for those with good internet access:

‘Before, I used to think of *** hospital as being an internet free zone, and now it hits your wallet a little bit, it’s a little bit expensive, but you know you can watch a lot of YouTube videos and yes there are a lot of podcasts around, so information is way more accessible.’ (BT5, CK, M5)

#### Subtheme 4: Formal and informal learning

Participants recognised and differentiated between formal and informal learning, the former being diplomas, degrees and courses that provided academic instruction and accreditation for career advancement. However, they also acknowledged the importance of informal learning that happened on the job and that was essential for improving service delivery because of its applied relevance:

‘I think there’s learning for accreditation and there’s learning for service delivery. I think they are quite different. Often courses are aimed at accreditation whereas outside of that, the doctor has other learning needs that are much bigger than that and should be ongoing.’ (MT6, VF, M9)

For participants, informal learning was dynamic, as it was any form of mentorship or self-directed learning by picking up things as they go about their daily business:

‘Informal learning occurs as you walk around, you observe, you meet a problem patient and want to find some information. These days we use Google, and Wikipedia and Emergency Medicine and other apps on your phone.’ (MT6, VF, M9)

The participants acknowledged the informal learning that takes place through exposure in a rural environment and that they learn from the patients and their surroundings. Some went as far as placing importance to what they learnt informally as it dealt with the immediate needs of the patient, with the formal learning being regarded as providing core knowledge:

‘So, I find the most effective way for me to learn, is when I see a patient and I realise that there is a gap in my knowledge or there is a gap in my experience and then I go research it, or find a guideline, or ask a senior on the spot. You learn something … I think the need to learn is created by the patients and I am the doctor, I am supposed to see them and manage them so then I experience the need to know and to learn.’ (MT3, BA, F4)

They also recognised that they could learn from everyone, and that engaging in peer learning or peer education is important in terms of how they learn:

‘[*N*]o matter how much bookwork you do, you’re never going to be confident in your skills, unless you’ve got somebody that is watching you and encouraging you, somebody that’s saying Yes, you’ve got it.’ (BT5, CK, M5)

This peer learning extended to formalised ward rounds and continuing medical education (CME) programmes organised by the hospital, with presentations on common conditions that everyone was expected to know and were likely to see in practice. These sessions encouraged group discussions and for them to interact and seek clarity on topics. This peer learning was further enhanced when there was senior support, as they identified the strength of learning from senior staff:

‘For me it’s easy to approach people if I have a learning gap. I can actually make a conversation with almost anyone, …, and it’s not just a matter of the people within my area of interest, it’s almost every staff member in the ward or anywhere in the hospital.’ (BT4, TN, M4)

‘[*I*]t’s always good to have someone senior to you, someone who you know that when you get stuck or where you feel I need some more brains to actually help me out, there’s always someone you can actually look up to or someone you can speak to.’ (BT4, TN, M4)

## Discussion

From the four subthemes identified regarding how they learn, it is evident that multiple influences need to align, the most obvious and well known being the level of embeddedness to clinical practice.^9^ The clinical practice they are exposed to, their own motivation and performance, and the way they engage in the learning process are all interlinked and have an impact on their continuous growth and transitioning in medicine, but these are also influenced by the environment they find themselves in.^27^ This means there needs to be some level of deliberate practice and continuous engagement in reflective practice as key principles for workplace learning,^28^ as it has been reported that reflecting on their experiences helps to improve professional practice and is regarded as a critical attribute towards lifelong learning for healthcare workers.^29^

Although learning must be deliberately pursued, there is evidence that shows that learning in the workspace is often informal and a by-product of work-based goals.^30^ A study done among Irish hospital doctors in Limerick, Ireland found that medical doctors of different grades had varying motivations to learn and used a variety of resources to inform their learning, from the traditional use of medical journals and textbooks, to consulting colleagues, and more recently, the use of technology, such as the internet. Participants in this study reported learning mostly by working with peers and senior colleagues, which highlighted the importance of clinical, on-the-job teaching and learning.^27^ This also highlights the importance of communities of practice, which, according to Wenger (1999)^31^ and Wenger et al. (2002), are social processes in which learning groups naturally form and interact with existing organisations to define members sense of identity and which promote learning.^28^

### Limitations of the study

While this study provides valuable insights into how medical doctors working in rural district hospitals understand their learning and learning needs, we have to acknowledge the possible limitations. This study was conducted in two rural district hospitals, which are located in the same district in rural KwaZulu-Natal, South Africa. This brings into question the generalisation of the findings to other rural district hospitals. The researcher uses to work in one of the hospitals utilised for the study, thus cannot exclude the possibility of bias in the interpretation of the findings. There is also the possibility of the Hawthorne effect impacting the study findings, as the researcher was known to the research environment as a specialist family physician. This may have also been impacted by the fact that the researcher was only able to spend 3 weeks physically in each hospital, while true exclusion of the possible Hawthorne effect requires one to fully immerse themselves into the research environment for prolonged periods. Despite these limitations, efforts were taken to minimise their effects by ensuring triangulation with the use of multiple tools for data collection. Future research, with more rural district hospitals in different provinces, and researchers who have minimal prior knowledge of the environment being studied could strengthen the authenticity of the research findings.

## Conclusion

### Why I learn, what I learn, how I learn

The aim of this study was to explore the views of medical doctors working in rural district hospitals, as to how they understand their learning and learning needs, thus further adding to the South African body of literature. According to educational psychologists, in order for learning to be successful, the learner needs to understand the content at a cognitive level, be willing to invest effort in studying at an affective level and have the ability to self-regulate their learning at a metacognitive level.^32,33,34^ This holds true at both undergraduate and postgraduate levels of education. In asking the question of how medical doctors understand their learning and learning needs, we needed to interrogate this through critically reflecting on their understanding of the affect (*why learn*), cognition (*what to learn*) and metacognition (*how to learn*) processes of learning, a concept well described by Ten Cate et al.^35^

The findings from the participants highlight the importance of individuals needing to be highly self-regulatory, especially in rural DHs that present multiple challenges for learning. They need to acknowledge their need for learning, as without an understanding of the need, without humility and acknowledging that one’s knowledge is incomplete, there is no incentive for ongoing learning.

This study has shown that to be self-regulatory, there needs to be a level of self-motivation, which can be explained by the self-determination theory, this being a theory of motivation that looks at a set of psychological mechanisms relating to an individual.^36^ It highlights the importance of individuals creating a sense of competence, autonomy in how they function, and relatedness to their surrounding environment.^32^ This was evident in our participants, who showed the importance of self-awareness, thinking of others (peers), considering the patient as a beneficiary of their learning and having consideration for external factors as they influence their learning.

We hope the findings from this study will strengthen how learning takes place and how to facilitate the creation of learning in rural district hospital environments. These insights might help medical educators and health care facility managers to better facilitate the learning needs of the staff, which in turn would contribute to the provision of quality health care.
